# Editorial: Omics Data Integration Towards Mining of Phenotype Specific Biomarkers in Cancers and Diseases

**DOI:** 10.3389/fcell.2021.763447

**Published:** 2021-10-15

**Authors:** Liang Cheng, Lei Deng, Chuan-Xing Li, Yan Zhang

**Affiliations:** ^1^NHC Key Laboratory of Molecular Probe and Targeted Theranostics, Harbin Medical University, Harbin, China; ^2^College of Bioinformatics Science and Technology, Harbin Medical University, Harbin, China; ^3^School of Computer Science and Technology, Central South University, Changsha, China; ^4^Respiratory Medicine Unit, Department of Medicine & Centre for Molecular Medicine, Karolinska Institutet, Stockholm, Sweden; ^5^The First Hospital of Lanzhou University, Lanzhou, China; ^6^Harbin Institute of Technology, Harbin, China

**Keywords:** multiple omics data, system biology method, molecular basis of disease, artificial intelligence, data integration

The development of high-throughput sequencing technology and the advent of omics approaches have been providing a solid basis for the systematic understanding of the function of human genes and the mechanism of cancers and other diseases. In the last years, the integration of multiple omics data has provided many advantages over single omics approaches in providing a more comprehensive understanding of the molecular basis of disease. For instance: the integration of genome-wide association studies (GWAS) data using Mendelian Randomization (MR) has been used widely in identifying causal phenotypes of human diseases; the integration of GWAS data and expression quantitative trait loci (eQTL) data using Summarized MR (SMR) can facilitate mining causal genes of human diseases; and the integration of microarray data and Next Generation Sequencing data using machine learning technology has been used to successfully identify gene signatures associated with clinically relevant molecular subtypes and prognosis in complex diseases. Though successful, the emergence of sequencing technologies such as single cell sequencing and metagenomics sequencing have posed more challenges for data integration methods. It is, therefore, essential to apply novel statistical methods and artificial intelligence approaches for integrating multiple omics data, such as single cell sequencing data, microbial Quantitative Trait Loci (mbQTL) data, and microbiome GWAS (mGWAS) data. Here, we organized a Research Topic on “System Biology Methods and Tools for Integrating Omics Data.” In total, 66 outstanding works were presented in this thematic issue, ten of which have been highlighted as follows.

- Yao et al. investigated the detection of severely ill patients with COVID-19 from those with mild symptoms using the clinical information and the blood/urine test data. Meanwhile they utilized the machine learning algorithms to build the COVID-19 severeness detection model. Support vector machine (SVM) demonstrated a promising detection accuracy after 32 features were detected to be significantly associated with the COVID-19 severeness. These 32 features were further screened for inter-feature redundancies. The final SVM model was trained using 28 features and achieved the overall accuracy 0.8148. This work may facilitate the risk estimation of whether the COVID-19 patients would develop the severe symptoms. The 28 COVID-19 severeness associated biomarkers may also be investigated for their underlining mechanisms how they were involved in the COVID-19 infections.
- Chang et al. focused on ceRNA and immune cells in colorectal adenocarcinoma (COAD). Meanwhile, they applied comprehensive bioinformatics methods to analyze differential expression genes (DEGs) related to metastasis and establish the ceRNA networks. The Cox analysis and Lasso regression were utilized to screen the pivotal genes and prevent overfitting. Based on them, the prognosis prediction nomograms were established. The cell type identification by estimating relative subsets of RNA transcripts (CIBERSORT) algorithm was then applied to screen significant tumor immune-infiltrating cells associated with COAD metastasis and established another prognosis prediction model. Ultimately, they found some significant ceRNAs (FAS and hsa-miR-125b-5p) and tumor-infiltrating immune cells (T cells follicular helper and Macrophages M0) might related to distance metastasis and prognosis of COAD. The constructed nomograms and the identified regulatory mechanism might provide new insights for the prediction and treatment of COAD metastasis in clinic.
- Song et al. elucidated the Lung adenocarcinoma (LUAD) immune heterogeneity to develop new immunotherapeutic strategies with better efficacy. Firstly, non-negative matrix factorization-based deconvolution was performed to identify robust clusters of 489 LUAD patients in The Cancer Genome Atlas (TCGA) and verify their reproducibility and stability in an independent LUAD cohort of 439 patients from the Gene Expression Omnibus (GEO). Then, they used the graph learning-based dimensionality reduction to visualize the distribution of individual patients. Next, they identified and validated four reproducible immune subtypes, Clusters 1–4 (C1–C4) associated with distinct gene module signatures, clinicopathological features, molecular and cellular characteristics. Finally, their investigations discovered a complex immune landscape with a scattered immune subtype profile. This work may help inform immunotherapeutic decision-making and design advanced immunotherapy strategies for the treatment of lung cancer.
- Huang R. et al. focused on breast cancer (BRCA), one of the most common malignancies in women. Firstly, they achieved datasets including RNA sequencing and alternative splicing events (ASEs) of BRCA samples from TCGA and TCGASpliceSeq databases. Then, a survival model was built including 15 overall-survival-associated splicing events (OS-SEs) by Cox regression and Lasso regression. The co-expressed splicing factors (SFs) of each bone-and-distant-metastasis-related OS-SE were discovered by Pearson correlation analysis. Additionally, Gene Set Variation Analysis (GSVA) was performed to identify the downstream mechanisms of the key OS-SEs. Finally, the results were validated in different online platforms. In brief, a reliable survival model was established, and CIRBP was found co-expressed with FAM110B associated with the fatty acid metabolism pathway. Meanwhile, they also proposed a potential molecular mechanism and therapeutic target of BRCA.
- Cancer patients often develop primary or acquired resistance to ICB and immune-related adverse events (IrAE). Therefore, Huang Q. et al. summarize the recent findings of internal and external causes of tumor resistance to immune checkpoint blockade (ICB). The internal causes focus on the inherent characteristics of tumor cells, such as tumor antigenicity, tumor escape mutation, interferon signal pathway, epigenetic, carcinogenic signal pathway, and so on; the external causes are mainly emanated from the tumor microenvironment, such as immunosuppressive cells, cytokines, metabolites, new immune checkpoints, intestinal microorganisms, and so on. Meanwhile, they also discuss the latest research progress in overcoming tumor resistance to ICB, in which combined immunotherapy stands at the center stage. In brief, Huang Q. et al. highlight tumor cell-intrinsic and -extrinsic factors that may underlie tumor resistance to immune checkpoint blockers. Targeting these factors in combination with immune checkpoint blockers points to the future direction of cancer immunotherapy.
- Xiao et al. focused on the mechanism underlying Gastric cancer (GC) occurrence and development. In particular, the role of lncRNAs in GC. In this study, they obtained and analyzed RNA sequencing (RNA-seq) data from 16 samples of eight gastric cancer patients. A total of 1,854 previously unannotated lncRNAs were identified by ab initio assembly, and 520 differentially expressed lncRNAs were validated in the TCGA expression dataset. Methylation and copy number variation (CNV) array data from the same sample were integrated in the analysis. Changes in DNA methylation levels and CNVs may be responsible for the differential expression of 91 lncRNAs. Differentially expressed lncRNAs were enriched in coexpressed clusters of genes related to functions such as cell signaling, cell cycle, immune response, metabolic processes, angiogenesis, and regulation of retinoic acid (RA) receptors. Finally, a differentially expressed lncRNA, AC004510.3, was identified as a potential biomarker for the prediction of the overall survival of gastric cancer patients.
- Min et al. understand the potential effect of the posttranslational modification on ovarian metabolic homeostasis and oocyte development potential in women with Polycystic ovary syndrome (PCOS). They carried out a quantitative analysis of acetylated proteomics in ovarian granulosa cells of PCOS and control groups by mass spectrometry. There was widespread lysine acetylation of proteins, of which 265 proteins had increased levels of acetylation and 68 proteins had decreased levels of acetylation in the PCOS group. Differentially acetylated proteins were significantly enriched in the metabolic pathways of glycolysis, fatty acid degradation, TCA cycle, tryptophan metabolism, and branched-chain amino acid degradation. Acetyl-CoA acetyltransferase 1 (ACAT1) was an enzyme central to these metabolic pathways with increased acetylation level in the PCOS group, and there was a negative correlation of ACAT1 acetylation levels in PCOS granulosa cells with oocyte quality and embryo development efficiency in the clinic. They demonstrated that lysine acetylation changes of key enzymes in PCOS granulosa cells attenuated their activities and altered metabolic homeostasis of the follicular microenvironment for oocyte maturation, which provided a new and important mechanism that regulated the ovarian metabolic disorders in PCOS.
- Nan et al. identified and verify the key genes and lncRNAs associated with acute lung injury (ALI) and explore the pathogenesis of ALI. Firstly, they identified differentially expressed lncRNAs between the ALI samples and normal controls using gene expression profiles. Then, they applied quantitative real-time PCR (qPCR) to detect the expression of MALAT1, microRNA (miR)-194-5p, and forkhead box P2 (FOXP2) mRNA in 1 μg/ml LPS-treated HPAEpiC. Next, MALAT1 knockdown vectors, miR-194-5p inhibitors, and ov-FOXP2 were constructed and used to transfect HPAEpiC. The influence of MALAT1 knockdown on LPS-induced HPAEpiC proliferation and apoptosis via the miR-194-5p/FOXP2 axis was determined using Cell counting kit-8 (CCK-8) assay, flow cytometry, and Western blotting analysis, respectively. Finally, the interactions between MALAT1, miR-194-5p, and FOXP2 were verified using dual-luciferase reporter gene assay. In brief, they demonstrated that MALAT1 knockdown alleviated HPAEpiC apoptosis by competitively binding to miR-194-5p and then elevating the inhibitory effect on its target FOXP2. These data provide a novel insight into the role of MALAT1 in the progression of ALI and potential diagnostic and therapeutic strategies for ALI patients.
- Zhao et al. identified Alzheimer's disease (AD)-related proteins in blood to help treatment and diagnosis. They proposed a hypothesis that similar diseases share similar proteins. Diseases with similar symptoms are caused by abnormalities of similar proteins. Therefore, they developed an iterative method based on disease similarity (IBDS). They combined Elastic Network (EN) with Minimum angle regression (MAR) to find the optimal solution. Finally, they used case studies and Summary data Mendelian Random (SMR) to verify this method. They selected 39 diseases which are highly related to AD. They correspond 1,481 kinds of proteins. In brief, Zhao et al. presented a novel method for prioritizing AD-related proteins. Seven proteins have tissue specificity in blood among these 284 proteins, which could be used to diagnose AD in future. Case studies and SMR have been used to prove the relationship between these 7 proteins and AD.
- Zheng et al. paid attention to the key molecules and mechanisms responsible for hypertrophy of the ligamentum flavum (HLF). They used an integrated transcriptome and proteomics analysis of human ligamentum flavum (LF), and subsequent immunohistochemistry and real-time PCR assays, to show upregulation of CRLF1 to be the dominant response to HLF. TGF-β1 significantly increased mRNA expression of CRLF1 through SMAD3 pathway. CRLF1 enhanced LF fibrosis via ERK signaling pathway at the post-transcriptional level and was required for the pro-fibrotic effect of TGF-β1. Knockdown of CRLF1 was shown here to reduce fibrosis caused by inflammatory cytokines and mechanical stress. Furthermore, they found that bipedal standing posture can cause HLF and upregulation of CRLF1 expression in mice LF. Overexpression of CRLF1 was indicated to cause HLF *in vivo*, whereas CRLF1 knockdown impeded the formation of HLF in bipedal standing mice. These results revealed a crucial role of CRLF1 in LF hypertrophy. They propose that inhibition of CRLF1 is a potential therapeutic strategy to treat HLF. In brief, they investigated the regulatory mechanism of CRLF-1 in HLF and explored the role of CRLF-1 *in vivo*.
- Cao et al. aimed to overcome epigenetic barriers to improve reprogramming efficiency and improve developmental rate in Somatic cell nuclear transfer (SCNT) embryos. They analyzed DNA methylation profiles of *in vivo* fertilized embryos and SCNT embryos with different developmental fates. Overall DNA methylation level was higher in SCNT embryos during global de-methylation process compared to *in vivo* fertilized embryos. In addition, promoter region, first intron and 3′UTR were found to be the major genomic regions that were hyper-methylated in SCNT embryos. Meanwhile, they found the length of re-methylated region was directly related to the change of methylation level. Furthermore, a number of genes including Dppa2 and Dppa4 which are important for early zygotic genome activation (ZGA) were not properly activated in SCNT embryos. In brief, this study comprehensively analyzed genome-wide DNA methylation patterns in SCNT embryos and provided candidate target genes for improving efficiency of genomic reprogramming in SCNT embryos.
- Cheng et al. focused on the molecular mechanisms underlying metastatic melanoma. They aimed to identify and validate prognostic biomarkers associated with metastatic melanoma. Firstly, they constructed a co-expression network using large-scale public gene expression profiles from GEO, from which candidate genes were screened out using weighted gene co-expression network analysis (WGCNA). A total of eight modules were established via the average linkage hierarchical clustering, and 111 hub genes were identified from the clinically significant modules. Next, two other datasets from GEO and TCGA were used for further screening of biomarker genes related to prognosis of metastatic melanoma, and identified 11 key genes via survival analysis. They found that IL10RA has the highest correlation with clinically important modules among all identified biomarker genes. Further *in vitro* biochemical experiments, including CCK8 assays, wound-healing assays and transwell assays, have verified that IL10RA could significantly inhibit the proliferation, migration and invasion of melanoma cells. Furthermore, gene set enrichment analysis showed that PI3K-AKT signaling pathway was significantly enriched in metastatic melanoma with highly expressed IL10RA, indicating that IL10RA mediates in metastatic melanoma via PI3K-AKT pathway.

Each study in the special issue was peer-reviewed by more than two external reviewers. We would like to thank all the authors for contributing their work to our hot topic issue and all the reviewers for their time and efforts. Finally, we would like to thank the Chief Editor and Editorial Office of Frontiers in Cell and Developmental Biology for their support during the whole processes.

## Author Contributions

LC and LD conducted this topic issue and wrote the manuscript. All authors contributed to the article and approved the submitted version.

## Funding

The Tou-Yan Innovation Team Program of the Heilongjiang Province (2019-15), National Natural Science Foundation of China (61871160 and 62172130), Young Innovative Talents in Colleges and Universities of Heilongjiang Province (2018-69), and Heilongjiang Postdoctoral Fund (LBH-Q20030).

## Conflict of Interest

The authors declare that the research was conducted in the absence of any commercial or financial relationships that could be construed as a potential conflict of interest.

## Publisher's Note

All claims expressed in this article are solely those of the authors and do not necessarily represent those of their affiliated organizations, or those of the publisher, the editors and the reviewers. Any product that may be evaluated in this article, or claim that may be made by its manufacturer, is not guaranteed or endorsed by the publisher.

